# New Generation of Clinical Epigenetics Analysis and Diagnosis for Precision Medicine

**DOI:** 10.3390/diagnostics15121539

**Published:** 2025-06-17

**Authors:** Pengtao Song, Biaoru Li

**Affiliations:** 1 Department of Pathology, Huzhou Central Hospital, Fifth School of Clinical Medicine of Zhejiang Chinese Medical University, Affiliated Central Hospital of Huzhou University, Huzhou 313000, China; song_pengtao@aliyun.com; 2 School of Medicine, Case Western Reserve University, 10900 Euclid Ave, Cleveland, OH 44106, USA; 3 Senior Faculty of Principal Research Scientist, Department of Pediatrics and GA Cancer Center, Children Hospital at GA, Augusta, GA 30913, USA

**Keywords:** epigenetics, epigenomics, in vitro diagnostics (IVD), single-cell diagnosis

## Abstract

Following the application of epigenetic and epigenomics research into tumor diseases, cardiovascular disease, diabetes, hereditary diseases, and rare diseases, in vitro diagnostics (IVD) epigenetic and epigenomics are increasingly employed for those patients. Here, we review a clinical sampling of epigenetics and epigenomics from patients. We then present procedures, including the detection procedure of clinical epigenetic approaches from clinical samples, clinical epigenomic methods applied to those samples, the small cell number of epigenetics, and epigenomics. Finally, we present the current IVD of epigenetics and epigenomics used for clinical analysis and diagnosis, along with the development of approaches. To improve clinical study and diagnosis, we also introduce clinical research and clinical applications to develop a more comprehensive strategy to maximize the sensitivity and specificity of the epigenetics and epigenomics analysis and diagnosis.

## 1. Introduction

Epigenetics is the study of abnormal gene expression by environmental change for human diseases and human development even though DNA sequences have not changed [1]. Now, epigenetics involves tumor diseases and non-tumor diseases such as cardiovascular disease (CVD), diabetes, hereditary diseases, immune diseases, infectious diseases, human behavior change, children’s growth, and neuron development in the brain [2,3,4,5,6,7,8,9,10]. Clinically, the epigenetics related to human diseases, human behavior, and children’s development is involved in three levels: DNA methylation, histone modification, and long non-coding RNA (lncRNA) regulating gene silence for a distinct gene expression change with the gene switch on/off by some external factors [11,12,13,14].

**Figure 1:**
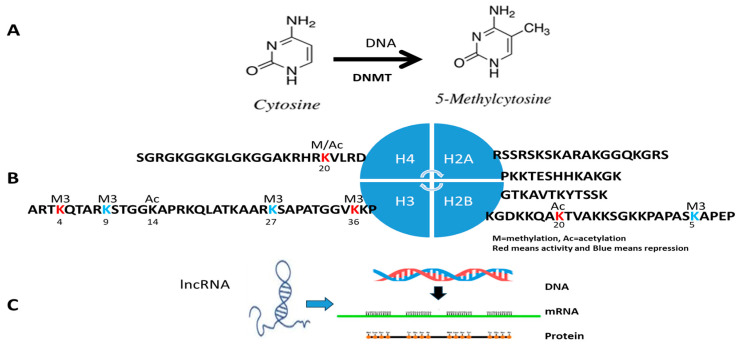
(**A**) **DNA methylation**. Methylation of cytosine is a covalent modification of DNA, in which hydrogen H5 of cytosine is replaced by a methyl group under DNA methyltransferase (DNMT). In mammals, 60–90% of all CpGs are methylated. The pattern of methylation controls protein binding to target sites on DNA, affecting changes in gene expression and in chromatin organization, often silencing genes, which physiologically orchestrates processes like differentiation and pathologically leads to cancer. (**B**) **Histone post-transcriptional modification**. Histones H2A, H2B, H3, and H4 are formed as the core histones with some long ‘tails’ located on one end for enzyme modifications, including methylation, acetylation, phosphorylation, and ubiquitination to adjust the regulatory proteins. The most well-understandable amino acids in histone in tumor diseases are H3K4Me3 and H3K36Me3 (activity, red color) and H3K9me2/3 and H3K27me3 (repressor, green color). K = lysine and R = arginine. (**C**) **lncRNA modification**.

First, epigenetics assays are to study DNA methylation at the C5 position of CpG dinucleotides with DNA methyltransferase (DNMT) activity. As Figure 1A shows, hypermethylation of CpG dinucleotides is often overexpressed in individual subjects associated with different diseases, while lower copying DNA methylation is passed down the methylated daughter strands during normal DNA replication. Therefore, DNA hypermethylation results in the silence of distinct genes at CpG sites in protein promoters. Following this, the DNMT family is increasingly studied; DNMT1 has been discovered to maintain existing DNA methylation patterns during DNA replication, while DNMT3A and DNMT3B are responsible for establishing de novo methylation patterns, which are new methylation patterns on previously unmethylated DNA. However, it is important to note that DNMT3A and DNMT3B also play a role in maintaining methylation patterns, and DNMT1 can also be involved in de novo methylation under specific circumstances [15]. Additionally, m6A methylation is a new modification where a methyl group is added to the sixth nitrogen atom of adenine, resulting in N6-methyladenosine (m6A). This modification is prevalent in eukaryotic RNAs, including mRNA, tRNA, rRNA, and non-coding RNAs, and plays a vital role in regulating various cellular processes. The methylation has been linked to various diseases, including cancer and age-related neurological disorders [16].

The second branch of epigenetics focuses on histone modification, as shown in Figure 1B. Histones consist of H2A, H2B, H3, and H4 in the core histones, and H1 and H5 are known as the linker histones in the five significant subtypes of the histone family [1,11,12]. The core histones are dimers with three alpha helices linked by two loops. The helical structure interacts with four distinct dimers to form one octameric nucleosome core, including two H2A-H2B dimers and an H3-H4 tetramer. The H2A-H2B dimers and H3-H4 tetramer are highly conserved with a ‘helix turn helix turn helix’ motif. Their long ‘tails’ are located on one end of the amino acid structure for enzyme modifications, including methylation, acetylation, phosphorylation, and ubiquitination to adjust the regulatory proteins with highly positively charged N-terminus at lysine (K) and arginine (R) residues. For example, tumor suppressor genes are silenced under hypermethylation in tumor diseases, as shown in Figure 1B [17,18].

The third focus of clinical epigenetics are RNA molecules, including lncRNA, miRNA, piRNA, and so on, that do not code for proteins. Instead, they play a crucial role in regulating gene expression, often by silencing specific genes through various mechanisms like binding to mRNA or influencing chromatin structure, as shown in Figure 1C. For example, lncRNAs are a group of longer than 200 nucleotides with four unique characteristics: lower quantity, higher tissue specificity, higher stage specificity, and higher cell subtype specificity. Clinical studies have find that lncRNAs are related to metabolism and immunity diseases and are closely related to the occurrence and development of tumors, CVD, nervous system disorders, and nephropathy [19,20].

**Figure 2:**
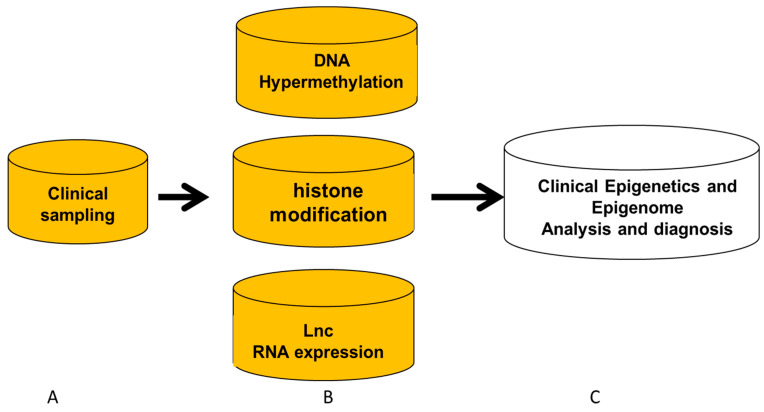
**Clinical epigenetic and epigenome analysis**. Process from sampling performance, epigenome and epigenome application for DNA hypermethylation, histone modification, and lncRNA for clinical epigenetic and epigenomic analysis and diagnosis. Pink means sampling and abnormal epigenetic changes, which will be discussed in detail in the manual.

Currently, clinical scientists and physicians focus on epigenetic analysis and diagnosis regarding human tumor diseases, CVD, diabetes, hereditary diseases, immune diseases, human behavior change, and children’s growth and development [2,3,4,5,6,7,8,9,10]. In this study, to introduce clinical epigenetic and epigenomics analysis and diagnosis in clinics, as shown in Figure 2, we will present (A) clinical sampling for epigenetic analysis and diagnosis of patients; (B) clinical epigenetics and epigenomics detection procedures with their analyses and diagnosis; and (C) research and development (R&D) of products and kits for epigenetics and epigenomics analysis and diagnosis.

## 2. Clinical Sampling for Epigenetics and Epigenomics

Abnormal epigenetic and epigenomic aberrances in genes play an essential role in disease formation and development by environmental change. Sampling for non-tumor diseases is mainly different from those for tumor diseases. To achieve optimal specimens for clinical epigenetics and epigenomic analysis and diagnosis, here we present two types of clinical sampling for epigenetic diagnosis: one from tumor-disease sampling from tumor patients and the other from patients suffering from non-tumor diseases, human behavior changes, and embryonic and children development.

1. **Clinical sampling from subjects with common diseases, human behavior changes, and development:** As shown in Table 1, tissue sampling is not a priority in conducting experimental studies of epigenetics and epigenomics for subjects with non-tumor diseases, human behavior, or children’s development. For instance, biopsies from the heart, pancreas, and brain tissues are very few because those tissues are difficult to acquire for clinical analysis and diagnosis. In human cardiovascular disease (CVD), diabetes, hereditary diseases, immune diseases, and human behavior change, the most common sampling is harvested from blood, saliva, cheek swabs, and follicles of patients, so we perform careful clinical analysis for the tissue specificity of epigenetic mechanisms related to this disease [21,22]. There are two ways to resolve the disadvantages of addressing epigenetics changes: (1) Physicians can try to achieve specific cells such as biopsy or isolate special cells to address specific epigenetics or epigenomics changes. As Table 1 shows, some sampling performance can increase specific epigenetics information, such as aorta biopsy to study CVD, isolating reticulocytes to study sickle cell disease or thalassemia, isolating lymphocytes or macrophages to study immune diseases, or isolating neutrophil for infectious diseases [10,23]. (2) Epigenetic analysis in silico can uncover some specific epigenetic change for patients. For example, we study epigenetic change related to mental health. In that case, the epigenetics results from blood specimens must co-study the variability in epigenetics from immune cells with their responses. Additionally, suppose we used buccal cells from saliva or cheek swabs for epigenetics study. In that case, these cells derived from the primitive germ layer (the ectoderm) may coincide with the epigenetic change of the brain to study children’s development and behavior [24,25].
2. **Clinical sampling from patients with tumor diseases:** As discussed above, tumor tissue sampling for specific epigenetic analysis is more advanced than that from subjects with non-tumor diseases. For example, we can isolate tumor cells from tumor tissues, separate tumor cells from circulating tumor cells (CTC), or culture primary tumor cells from tumor tissues in a clinical laboratory [12,26].

(1) **Isolating tumor cells from tumor tissue, CTC, and cultured tumor cells**

Clinical sampling for the isolation of tumor cells from patients includes flow-cytometric cell sorting (FACS), magnetic cell separation (MACS), and laser-captured micro-dissection (LCM) with downstream epigenetic analyses analysis [27]. FACS can isolate tumor cells or cancer stem cells (CSC) by a specific biomarker such as EpCAM on the tumor cell surface. The MACS technique of clinical sampling is often used to sort CSCs using cell-surface biomarkers, such as CD133/CD34 for CSCs. Laser-capture microscopy (LCM) has very good advantages, relying on tumor cell morphology with the change in the arrangement of glass slides in an in vivo environment. After cell staining (IHC/ICC) and DNA/RNA-based staining, LCM techniques are increasingly used in patients with tumor disease because of clinical pathologists’ assistance. CTCs are cells shed into the blood from a primary tumor location or metastatic tissues. Tumor cells have been increasingly detected in circulation blood from breast, prostate, lung, and colorectal cancer patients. We have been working on enriching the clinical cells of tumor patients and genetic disease patients for more than 25 years [28]. For example, isolating CTCs from withdrawing 10 mL blood is a fundamental performance under negative selection (CD45)/positive selection (for example, CD326) called enrichment-CTC. Cultured primary tumor cells have been reported for drug sensitivity assay in our laboratory since 1994. Now, we have routinely used techniques to increase primary cell numbers with downstream clinical genomic analyses and drug screening so that tumor cell cultures from clinical specimens with downstream epigenetic analyses can be used [27,28].

(2) **Liquid tissue**

Liquid tissue sampling includes two kinds of specimens: cell-free DNA (cfDNA) from body fluid and exosome for epigenetic analysis. DNA from body liquid sampling is a collection of DNA fragments released from any dying cell in the body fluid, while exosome DNA from body liquid is specifically found DNA within exosomes secreted by living cells, meaning that exosome DNA is a subset of materials providing more specific information from living cell secretion. The exosome DNA is considered a more targeted material within body fluids compared to DNA from body liquids, such as urine, sputum, bronchoalveolar lavage (BAL), mammary aspiration fluid, saliva, and stool. When tumor cells are degraded within a fluid, as above, DNA can be directly extracted. Because exosomes secrete from living cells into the fluid as above, both DNA and RNA extracted from exosomes can be harvested for epigenetics analysis and diagnosis. The cfDNA can be analyzed from frozen and fresh plasma, while the disadvantages are a lower abundance of cfDNA so that exosome can be developed to detect aberrant genes in DNA or RNA change in epigenetic analysis and diagnosis as described above, especially in patients with the condition of cell lysis or blood coagulation. Histones and histone modifications provide epigenetics information at chromatin states for gene transcription and translation. Chromatin immunoprecipitation (ChIP) is an antibody-based technology used to selectively enrich specific DNA-binding proteins along with their DNA targets. Now, frozen tissues can be increasingly reported histone modifications for epigenetics information at the level of tumor tissue. Samples from patients for lncRNA analysis and diagnosis either come from tumor tissue or from body fluids. As per our report, lncRNA sampling from body liquid specimens is majorly observed from CTC and exosome [29,30].

## 3. Detection Procedure of Clinical Epigenetics and Genomics

After clinical sampling is subject to the process for epigenetics, we need to consider downstream performance such as DNA methylation, histone modification, and lncRNA. All selections are based on three factors: (1) What is the clinical purpose of epigenetics analysis and diagnosis? For example, it is for clinical prediction, prevention, prognosis, personalized therapy, or others? (2) What kind of epigenetics changes are more related to subjects? and (3) whether or not a specific assay from tissues/cells is required or a general detection from a blood source. For example, specific cells/tissues are available to support personalized medicine for patients, while the liquid source is to support clinical prediction, prevention, and prognosis. Moreover, if a physician needs to longitudinally monitor prognosis, blood, saliva, urine, or follicles is the first choice because it is easily accessible and minimally invasive to achieve blood, saliva cheek cells, or urine.

1. **Clinical IVD for DNA methylation and DNMT**

**Figure 3:**
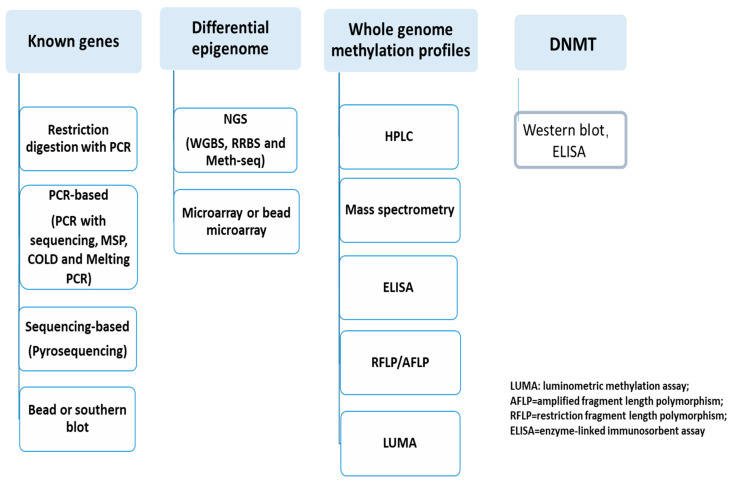
**Hypermethylation detection technique**. All technologies to detect DNA methylation are classified: Local specific genes which have been successfully employed in both research lab and clinical fields; Genomic regions and global measurement at a genome-wide scale based on sequencing technology and microarray, which have been successfully used in both research and clinical fields; Global measures of DNA methylation, which are often applied for research lab or clinical lab with equipment; and DNMT assay.

Quantifiable DNA methylation and DNMT detection are very important clinical application techniques. Currently, technologies for detecting DNA methylation have been widely applied in clinical research over the past 35 years of development, as shown in Figure 3. Clinically, feasible results of clinical diagnosis depend on the choice of methodology, including (A) the amount and quality of the DNA sample, such as DNA from formalin-fixed paraffin-embedded (FFPE) or from a liquid biopsy; (B) a method of sensitivity and specificity for clinical epigenetics; or (C) the robustness and simplicity of the assay method and the availability of specialized equipment, reagents, and bioinformatics software. The methylation and DNMT analysis and diagnosis include (I) specific known gene measures of DNA methylation, (II) differential epigenetics, (III) whole genome methylation, and (IV) DNMT enzyme assay, as shown in Figure 3. In clinical applications, DNA hypermethylation includes known-gene detection, unknown-gene or genomic hypermethylation, and DNMT assay [31,32,33,34].

(1) **Known-genes epigenetics**

If we know the DNA epigenetic aberrance in some clinical diseases, the techniques include PCR-based, pyrosequencing, restriction enzyme digestion assay, and Southern blot. Because restriction enzyme digestion and Southern blot are not often used in clinical laboratories, the two techniques are very often used with available products from companies.

**PCR-based methylation detection** includes methylation-specific PCR and PCR-based sequencing in clinical fields, while High-Resolution Melting and COLD-PCR are shown in Figure 3 [1,11,12]. For example, methylation-specific PCR uses bisulfite-converted DNA with methylation-specific PCR by two pairs of primers are designed: one pair amplifies methylated DNA, and another amplifies unmethylated DNA. Two qPCR reactions are performed for each sample, and thus, relative methylation is calculated based on the difference in their Ct values. The program for the design of methylation-specific primers can be found at (http://www.urogene.org/methprimer, accessed on 12 June 2025). PCR-based sequencing is first performed PCR in which primers are designed around the CpG island (MethPrimer software at http://www.urogene.org/methprimer, accessed on 12 June 2025), and thus used for PCR amplification of bisulfite-converted DNA. The resulting PCR products could be sequenced in the methylation status of individual CpG sites within the CpG islands of interest [35].

**Sequencing-based methylation detection** is pyrosequencing designed or purchased from PyroMark CpG Assays from Qiagen. After PCR products are obtained, a short-read pyrosequencing reaction (~100 bp) is performed. Because signal intensities for incorporated dGTP for methylated CpC and dATP for unmethylated DNA are increased, the level of methylation for each CpG site is quantified within the sequenced region, and the technique can assay very small aberrance in methylation (<5%). It is a good technique for heterogeneous tumor samples in which only a fraction of cells in mixed cells have a differentially methylated gene aberrance. Pyrosequencing requires some equipment to detect, such as the Qseq instrument from Bio Molecular Systems or PyroMark from Qiagen [36].

(2) **Unknown genes or epigenomic aberrance**

Although we had reported random restriction enzyme digestion with fingerprint to discover aberrance methylation in the precancerous tissue, now we use the genomic technique to uncover hypermethylation in the new tumor suppressor genes. Current methods for the identification of differentially and unknown methylated regions include next-generation sequencing (NGS) and microarray techniques [37].

**NGS-based detection of epigenomics** is bisulfite sequencing to assay DNA methylation aberrance. Because the bisulfite process can transform cytosine into uracil and then convert it to thymine, 5mC residues are resistant to this conversion, and thus methylated DNAs remain cytosine initially and then transform into guanine. Therefore, sequencing read from a pair of untreated DNA samples and bisulfite treated in the same sample enables the detection of the methylated cytosine. This approach has now been developed into whole genome bisulfite sequencing (WGBS). To increase the sequencing coverage for differentially methylated regions, anti-methylcytosine binding proteins (MBD) or antibodies against 5mC (MeDIP) have been used to enrich methylation DNA. NGS also developed reduced representation bisulfite sequencing (RRBS) to increase coverage. Sequencing could be done using any available NGS platform, such as Illumina and Life Technologies, with isolation of ~85% of CpG islands in the human genome performed as for WGBS; at the same time, the RRBS procedure normally requires about 0.1 ug~1 ug of DNA after successful MspI digestion. CpG-rich regions or specific regions from Agilent can use bait sequences to hybridize immobilized oligonucleotides. Such products are commercially available, called SureSelect Human Methyl-Seq from Agilent with targeted bisulfite sequencing (Methyl-seq) [38].

**Array-based detection** is a methylated DNA of the genome obtained by immunoprecipitation that could be used for hybridization by microarrays. At present, at least three companies support microarray chips or beads, such as the Human CpG Island Microarray Kit from Agilent, the GeneChip Human Promoter 1.0R Array, and the GeneChip Human Tiling 2.0R Array Set from Affymetrix. These arrays can use bisulfite-converted DNA to detect DNA methylation within gene promoter regions, enhancer regulatory elements, and 31 untranslated regions (31UTRs). The Infinium HumanMethylation450 Bead Chip from Illumina can detect 485,000 individuals CpG from 0.5 ug input DNA in 99% known genes. Technically, bisulfite-treated genomic DNA is mixed with assay oligos; one is complimentary to uracil from the original unmethylated cytosine, and another is complimentary to the cytosine of the methylated site. With hybridization, labeled assay oligos are immobilized to bar-coded beads, and the signal is measured for its methylation level [39].

(3) **DNMT measurement**

DNMT methods measure DNA methyltransferase (DNMT) activity and inhibition, which can be used to diagnose tumor and non-tumor diseases for a variety of methods, such as Western blot, ELISA-based assays, colorimetric assays, and fluorescence assays. Western blot characterizes the DNMT1 quantitation and activity. A colorimetric assay uses a microplate and equipment to measure the enzyme activity. ELISA-based assays use the ability of methyl CpG binding domain (MBD) proteins to bind methylated DNA quantified by spectrophotometry. Fluorescence assays are to use a self-assembly nucleic acid probe signal amplification to detect DNMT1 activity [40,41,42].

2. **Clinical IVD procedure for histone modification**

**Figure 4:**
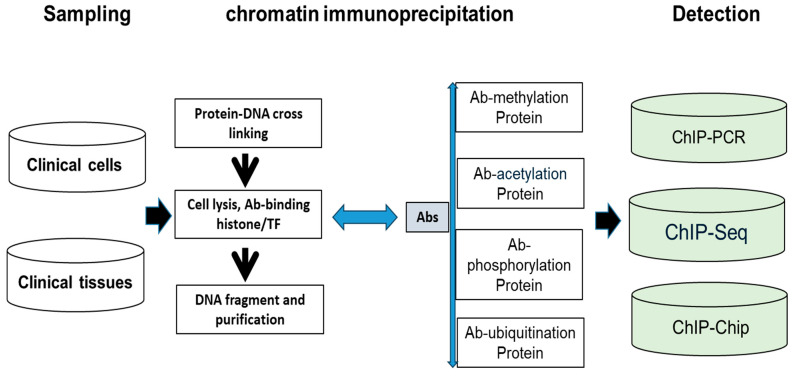
**Abnormal histone detection technique**. First step is sampling, including cell level and tissue level. Second step is the ChIP process, including crossing-binding, cell lysis, Ab-binding, and DNA purification. Third is the detection step, including ChIP PCR, ChIP-chip, and ChIP-seq.

IVD histone modifications have been increasingly reported in patients’ specimens from clinical cells and tissues. This protocol consists of two steps: (A) chromatin immunoprecipitation of crosslinking proteins-DNA (ChIP); (B) lysis cells with the crosslinking proteins-DNA, DNA fragment, and DNA purification. As Figure 4 shows, abnormal histone modifications include acetylation, methylation, phosphorylation, and ubiquitination so that special Ab can detect abnormal histone to differentiate the histone change between tumor cells and normal cell control. Finally, the purified DNA can be analyzed by qPCR (chromatin immunoprecipitation PCR, ChIP-PCR), DNA microarrays (ChIP-chip with downstream microarray), or Chip-seq (ChIP with downstream NGS). At present, ChIP-seq has been successfully used in formalin-fixed paraffin-embedded (FFPE) tissue. Moreover, histone modifications (PTM) detection by mass spectrometry (MS) has been used in clinical patients [43,44,45,46].

3. **Clinical IVD procedures for lncRNA**

**Figure 5:**
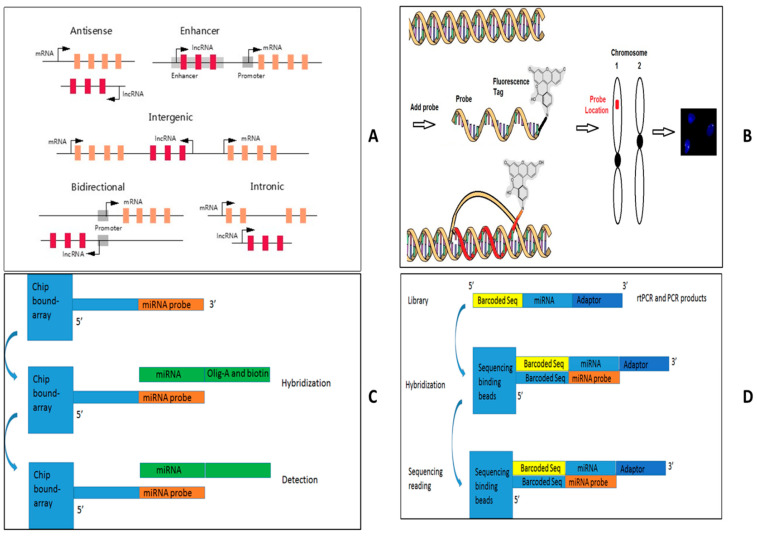
**Abnormal lncRNA detection technique**. (**A**) Specific lncRNA detection by either real-time PCR, (**B**) lncRNA FISH, (**C**) genomic detection by microarray, and (**D**) next-generation RNA sequencing.

There are four methods to detect lncRNA expressions such as Figure 5: (A) specific lncRNA detection by either real-time PCR, (B) lncRNA FISH, (C) genomic detection by microarray, and (D) next-generation RNA sequencing. Because lncRNAs can be classified into antisense, intergenic, intronic, overlapping, and bidirectional according to the position and direction of transcription in relation to other genes, some companies set up lncRNA databases with their primer designs for Q-rtPCR as Figure 5A [47,48,49,50]. For example, QIAGEN set up an in-house database based on human Gencode 19 with the confirmed lncRNA databases. RNA FISH is a cytogenetic technique that uses a fluorescent probe to bind to lncRNA with a high degree of sequence complementarity, as shown in Figure 5B. A fluorescence microscope can find out where the fluorescent probe is bound to the lncRNA. Because lncRNA is only expressed at the transcriptional level without protein expression, lncRNA FISH will be a very important technique to define the lncRNA expression within cells and tissues, though RNA FISH can be used to detect all three RNA (mRNA, miRNA, and lncRNA). Some companies have routinely serviced a probe design with their detection system [51,52,53,54].

In microarray-based approaches, as shown in Figure 5C, two screening methods, traditional microarray and tiling array, are used to identify lncRNAs. Because traditional microarrays can only detect the presence or absence of known lncRNAs in an RNA pool, they cannot detect novel lncRNAs. DNA tiling arrays contain oligonucleotide probes encompassing an entire length of a defined DNA region to identify novel lncRNAs, so DNA tiling arrays are a significant advantage in discovering new lncRNA. Some companies developed microarray chips to detect lncRNA Array star and Affymetrix, with more than 30,000 lncRNA expression panel assays [55,56,57,58]. RNA-seq is a potent technique to detect and quantify lncRNAs, as shown in Figure 5D. Because the disadvantage of an RNA-seq is the time and cost of the downstream analysis of the data, it is only used to discover previously unknown lncRNAs. Now, after the removal of rRNA from total RNA, which is suggested by some commercially available kits, both polyadenylated RNA and non-polyadenylated RNA can be used for lncRNA-seq. The reads are used to assemble a transcriptome and discover previously unannotated transcripts. Novel lncRNAs can be identified by excluding protein-coding transcripts and annotated lncRNAs based on the databases of RefSeq, ENCODE, and FANTOM (Functional Annotation of the Mammalian Genome). The selection of the four methods relies on clinical purpose. For example, if lncRNA is assayed as one or two lncRNAs, qPCR assay and RNA FISH are the first options. lncRNA-tiling array and RNA-seq will be very good candidates for clinical scientists to screen new lncRNA alteration, while traditional microarray can detect known lncRNA. Finally, lncRNA profile performance should also be considered in downstream analysis by skilled bioinformatics scientists and available tools [59,60,61,62,63,64].

## 4. R&D of Clinical Epigenetics Diagnosis

1. **Technology development**

The detection technology of abnormal DNA methylation, abnormal histone modification, and abnormal lncRNA has been largely developed so that epigenetics and epigenomics analysis and diagnosis have been increasingly applied to patients. Here, we present two R&D for epigenetic diagnosis: one is a single-cell technique to detect abnormal epigenetics, and the other is a new enzyme discovery for abnormal histone modification.

**Figure 6:**
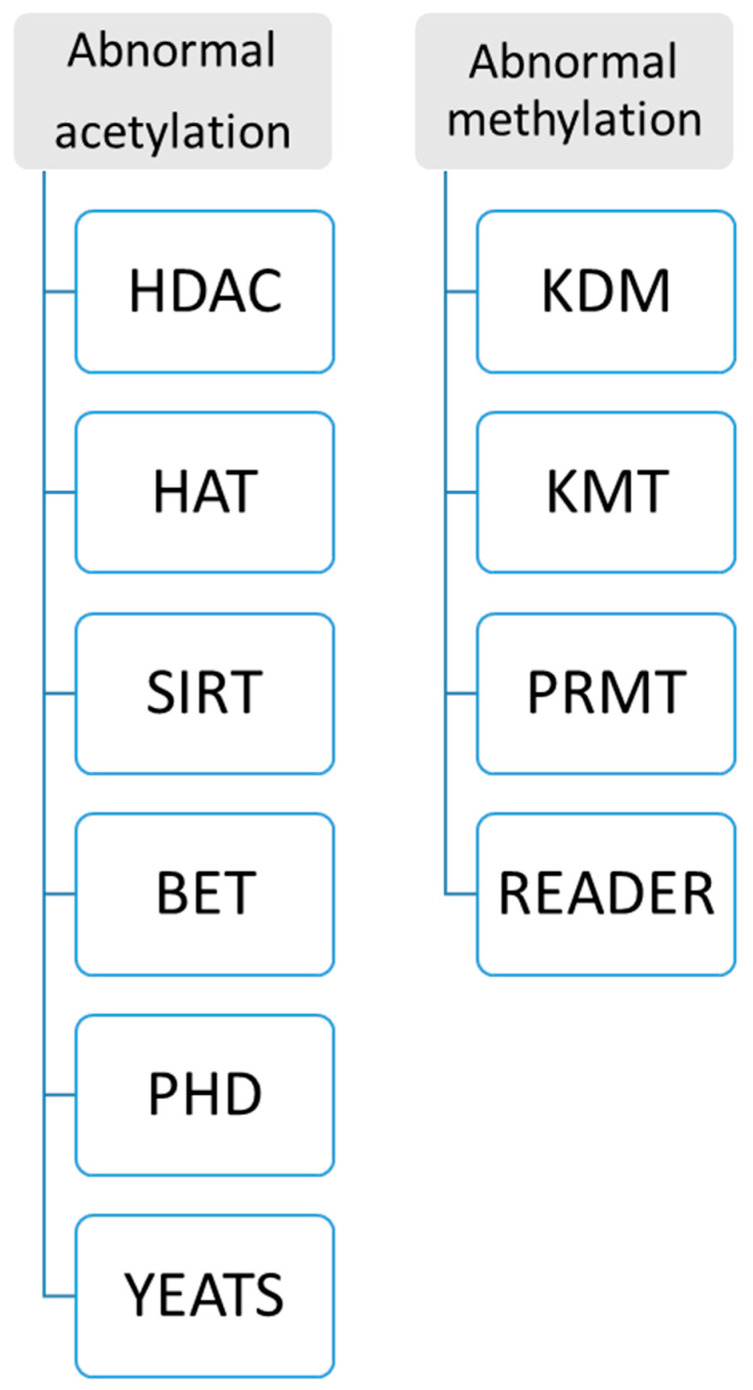
**Abnormal histone confirmation technique**. After abnormal histone modification is discovered, two kinds of confirmation assays for abnormal histone modification have been used in clinical patients: abnormal acetylation histone modification and abnormal histone methylation modification.

(1) Single-cell technologies enable profiling DNA methylation (DNAm) at cytosines in individual cells with a bioinformatic program, a transformer-based deep learning model for inputting DNAm states at each CpG site for single-cell analysis. Moreover, single-cell lncRNA technologies have been successfully used in animal models, so the techniques will be developed to detect lncRNA for clinical specimens from patients [65,66].
(2) New enzyme and protein domains discovered in epigenetics change can support precision medicine. Because abnormal histone modification is a very complicated mechanism, a novel R&D technique should be developed by different enzymes and targeting domains. Currently, two kinds of assays for abnormal histone modification have been used in clinical patients as Figure 6: (A) abnormal acetylation histone modification and (B) abnormal histone methylation modification. For example, abnormal histone acetylation with the enzyme or domain has been discovered to develop detection of HDAC (histone deacetylase), SIRT (class III histone deacetylases) activity, KAT activity (lysine acetyltransferase enzymes), BET (bromodomain and extra-terminal involved in acetylated histones) assay, plant homeodomain (PHD) finger detection, YEATS (domain acylated histones). Abnormal methylation, as shown in Figure 6, includes lysine methyltransferases (KMTs) and demethylases (KDMs), and Protein Arginine Methyltransferase (PRMT). Now, more and more enzymes and methods such as acetyltransferase 1 (HAT1), General Control Non-Repressible 5 (GNC5), CREB-binding protein (CBP), P300/CBP-binding protein (PCAF), MYST family, P300, TAFII250, and Rtt109 are increasingly reported in histone modification, which can increase clinical epigenetics analysis and diagnosis to increase epigenetics therapeutic potential [67,68,69,70,71,72,73,74].
2. **Product development**

Based on technique development as described above, epigenetic and epigenomic analysis and diagnosis kits for abnormal DNA methylation, abnormal histone modification, and abnormal lncRNA expression have been increasingly reported that can increase clinical applications. For example, a growing aging population requires product development in the application of epigenetic treatments for senior patients. The rising requirement will encourage companies to develop new advanced kits for epigenetics or epigenomic diagnosis [75].

3. **Clinical development**

Based on the R&D of techniques and products, epigenetics and epigenomics analysis and diagnosis have been largely developed for tumor diseases and non-tumor diseases. As reported by the American Cancer Society, new tumor patients were 1.9 million, with around 609,360 deaths in the U.S., so most of them need epigenetics analysis and diagnosis. On the other hand, the non-tumor application of epigenetics and epigenomics have increasingly been reported, including for Alzheimer’s disease, Parkinson’s disease, CVDs, and diabetes, so some of them need clinical epigenetics analysis and diagnosis for patients [76].

## 5. Conclusions

Clinical epigenetics and epigenomics analysis and diagnosis is a new clinical field to assay abnormal DNA methylation, abnormal histone modification, and abnormal lncRNA in our clinical diagnosis fields. We have been studying epigenomic and epigenetic techniques for precision medicine for almost twenty years [11]. Although epigenetic mechanisms are largely reported as our published books [1], if we used epigenetics therapy as precision medicine, two questions still need to be be addressed: (A) clinical epigenetic analysis and diagnoses require a report with specificity and sensitivity test; and (B) clinical epigenetic analysis and diagnoses require a more effective confirmation test, such as oncogene domain-inhibiting confirmation, after a new generation of abnormal epigenetic mechanisms, such as histone modification, are developed.

Because the clinical epigenetics analysis can be increasingly applied to patients, after epigenetic screening and confirmation methods are developed for diagnosis, FDA-approved compounds can be used for targeted therapy at the bedside (which we will publish separately, such as epigenetic treatment concepts, epigenetic regimen, personalized epigenetic therapy, and optimizing combinations of epigenetic and genetic modification) so that detection of abnormal epigenetics change should greatly improve the treatment efficacy of epigenetic therapy and precision medicine in the future.

## Figures and Tables

**Table 1:** Epigenetic and epigenomic specimens for non-tumor diseases.

**Diseases**	**Common Sampling**	**Specific Sampling**
CVD	blood (such as WBC), and saliva, urine, follicles	Tissues affected areas (such as biopsy of the aorta) [23]
Diabetes	blood (such as WBC), and saliva, urine, follicles	Tissue biopsy for affected areas
Immune diseases	blood (such as WBC), and saliva, urine, follicles	Tissue biopsy for affected areas
Infection disease	blood (such as WBC), and saliva, urine, follicles	Tissue collection
Hereditary diseases	blood (such as WBC), and saliva, urine, follicles	Pathological tissues such as SCD reticulocyte
Children behavior	blood (such as WBC), and saliva, urine, follicles	N/A
Children development	blood (such as WBC), and saliva, urine, follicles	N/A

## Data Availability

Not applicable.
